# Strategies to Manage Dosing Deviations and Interruptions of Cabotegravir Long‐Acting Intramuscular Injections

**DOI:** 10.1002/cpdd.1568

**Published:** 2025-07-11

**Authors:** Kelong Han, Ronald D. D'Amico, William R. Spreen, Susan L. Ford

**Affiliations:** ^1^ Clinical Pharmacology Modeling and Simulation GSK Collegeville PA USA; ^2^ Clinical Development ViiV Healthcare Durham NC USA; ^3^ Clinical Pharmacology Modeling and Simulation GSK Research Triangle Park NC USA

**Keywords:** cabotegravir, dosing deviations, dosing interruptions, dosing strategies, HIV, pharmacokinetics, simulation

## Abstract

Long‐acting cabotegravir is approved for HIV treatment and prevention. To guide management of dosing deviations and interruptions, concentration‐time profiles for monthly and every 2 months regimens were simulated using a population pharmacokinetic (PPK) model. Adequate exposure was defined as trough concentration (C_tau_) >0.45 µg/mL (observed 5th percentile of first C_tau_ in pivotal studies) in >95% of subjects and maximum concentration (C_max_) <13.1 µg/mL (highest observed median steady‐state C_max_ in previous studies) in >50% of subjects. Simulations showed: (1) median C_max_ remained ≤6.35 µg/mL after doubled doses; (2) C_tau_ was suboptimal after half dose at first injection but recovered with a corrective dose; (3) injection delays ≤7 days maintained adequate C_tau_, while longer delays caused extended low‐exposure periods (≤23 days for 1‐month delay, ≤83 days for 3‐month delay); (4) reinitiating loading dose after delays >1 month led to higher exposure than continuing injections and may mitigate efficacy loss and resistance risks; and (5) oral bridging (30 mg daily) maintained adequate exposure during delays. Recommended strategies include no action for higher‐than‐planned doses, corrective dosing for lower‐than‐planned doses, strict adherence to schedule, reinitiating the loading dose after delays >1 month, and oral bridging. These findings were incorporated into product labeling and can inform next‐generation cabotegravir and other long‐acting agent development.

Key strategies in controlling the human immunodeficiency virus (HIV) epidemic include sustained virologic suppression at undetectable levels in people with HIV and prevention of acquisition through pre‐exposure prophylaxis (PrEP) in individuals likely to be exposed to HIV. Although daily oral antiretroviral therapies are highly effective and generally well tolerated, they are associated with several limitations,^1^, ^2^, ^3^ including challenges to stay adherent for some individuals, burden and anxiety of daily dosing, inadvertent disclosure to others, negative stigma, and daily reminder of HIV status. Availability of long‐acting (LA) agents may overcome challenges associated with daily oral dosing.^4^, ^5^

Cabotegravir (CAB) is a small‐molecule HIV integrase strand transfer inhibitor.^6^, ^7^, ^8^, ^9^, ^10^, ^11^ The combination of CAB LA and rilpivirine (RPV) LA, administered by a healthcare professional monthly (QM)^12^, ^13^ or once every 2 months (Q2M)^14^ via intramuscular (IM) gluteal injections, is a complete LA regimen approved for maintenance of HIV‐1 virologic suppression in people with HIV (CABENUVA and VOCABRIA). Cabotegravir LA, administered Q2M as a single agent (APRETUDE), was approved for HIV‐1 PrEP.^15^, ^16^ In addition, CAB oral tablet formulation (VOCABRIA) is approved as an optional short‐term lead‐in to assess tolerability^17^ and as a short‐term bridging for CAB LA dosing interruptions.

Dosing deviations of CAB LA are uncommon in CAB clinical studies,^12^, ^13^, ^14^, ^15^, ^16^ but they can occur due to factors such as incomplete injection (eg, seepage from the injection site and necessary interruption of injection) or the administration of an incorrect vial, as both 2‐mL (400‐mg) and 3‐mL (600‐mg) vials are commercially available. Dosing deviations may or may not be identified at the time of the injection with the incorrect dose, and corrective actions may or may not be needed. Dosing interruptions or delays of CAB LA may also occur because the injections are administered by healthcare professionals in clinic settings, while the recipients may miss the scheduled clinic visit or be unavailable for an extended period of time due to planned events (eg, scheduled travel) or unforeseen circumstances (eg, accidents, bereavement, or loss to follow‐up).

Management of dosing deviations and interruptions of CAB LA is crucial and must be addressed in the prescribing information to guide clinical practice. The objective of this analysis was to develop CAB dosing strategies using population pharmacokinetic (PPK) simulations to manage dosing deviations, unplanned dosing delays, and planned dosing delays with or without oral bridging.

## Methods

### Scenarios of Dosing Deviations and Interruptions

**Figure 1:**
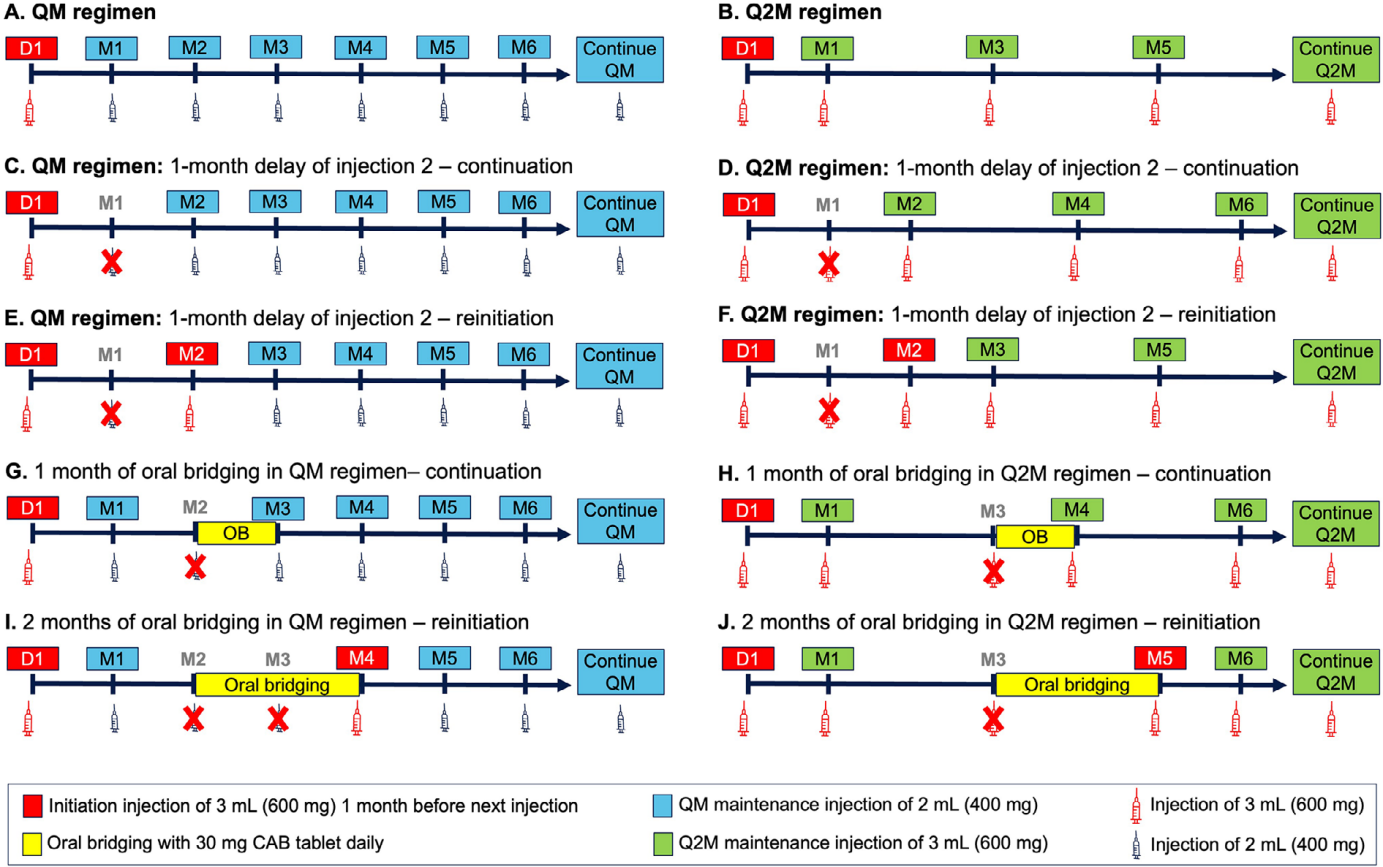
Scenarios of dosing deviations and interruptions of cabotegravir long‐acting injections. (A, B) The approved QM (A) and Q2M (B) regimens. (C‐F) Resumption of the 2nd injection of the QM (C and E) and Q2M (D and F) regimens after a delay of 1 month with continuation of regular QM (C) or Q2M (D) maintenance injections on resumption or reinitiation of the QM (E) or Q2M (F) regimen on resumption. Continuation was defined as resumption of regular maintenance dose (2 mL QM or 3 mL Q2M) after the delay. Reinitiation was defined as resumption with a 3‐mL initiation injection, followed by 2 mL QM (QM regimen) or 3 mL Q2M (Q2M regimen) starting 1 month after the 3‐mL initiation injection. (G‐J) Oral bridging for 1 month with continuation on resuming the delayed injection (G and H) or 2 months with reinitiation on resuming the delayed injection (I and J) in the QM (G and I) and Q2M (H and J) regimens. Oral bridging consisted of cabotegravir 30 mg oral tablet daily from the day of the missed injection until the day of the resumed injection. Continuation was defined as resumption of regular maintenance dose (2 mL QM or 3 mL Q2M) after the delay. Reinitiation was defined as resumption with a 3‐mL initiation injection, followed by 2 mL QM (QM regimen) or 3 mL Q2M (Q2M regimen) starting 1 month after the 3‐mL initiation injection. Scenarios for the QM (A, C, E, G, and I) and Q2M (B, D, F, H, and J) regimens. D, day; M, month; OB, oral bridging; QM, monthly; Q2M, once every 2 months.

Dosing of CAB LA in the approved QM and Q2M regimens starts with an initiation injection (loading dose) of 3 mL (600 mg), followed by maintenance injections of 2 mL (400 mg) QM (for the QM regimen) or 3 mL (600 mg) Q2M (for the Q2M regimen) starting 1 month after the initiation injection (Figure 1A,B). The steady‐state injection in this analysis was defined as either the 11th injection of the QM regimen or the 6th injection of the Q2M regimen because steady state is achieved within 1 year of CAB LA dosing.^18^

To evaluate the impact of higher‐than‐planned doses, the extreme scenarios of doubled doses were simulated: 6 mL for the initiation injection (instead of 3 mL), 4 mL for the steady‐state injection in the QM regimen (instead of 2 mL), and 6 mL for the steady‐state injection in the Q2M regimen (instead of 3 mL). Recommendations based on the steady‐state injections are applicable to all other injections because steady‐state exposure is the highest.

To evaluate the impact of lower‐than‐planned doses, the extreme scenarios of half dose were simulated: 1.5 mL for the initiation injection (instead of 3 mL), 1 mL for the 2nd injection of the QM regimen (instead of 2 mL), and 1.5 mL for the 2nd injection of the Q2M regimen (instead of 3 mL). A corrective injection with the originally planned dose was administered 2 weeks after the incorrect dose. Subsequent injections were simulated under 2 scenarios: (1) maintaining the original injection dates, that is, subsequent injections starting 1 or 2 months after the incorrect dose, or (2) resetting the injection dates, that is, subsequent injections starting 1 or 2 months after the corrective injection. Recommendations based on the first 2 injections are applicable to all other injections because exposure following the first 2 injections is the lowest.

To evaluate the tolerable injection window, the extreme scenario was simulated where every injection of the Q2M regimen was delayed by up to 7 days. Recommendations based on the Q2M regimen are applicable to the QM regimen because exposure following the QM regimen is similar to or higher than that of the Q2M regimen.^18^

Delays of the 2nd injection by 1‐12 weeks were simulated for both the QM and Q2M regimens. To evaluate the recovery of CAB exposure after resuming the delayed injection, subsequent injections were simulated under 2 scenarios: (1) continuation (Figure 1C,D), where the regular maintenance dose (2 mL QM or 3 mL Q2M) was resumed after the delay, or (2) reinitiation (Figure 1E,F), where a 3‐mL initiation injection (loading dose) was resumed, followed by 2 mL QM (QM regimen) or 3 mL Q2M (Q2M regimen) starting 1 month after the 3‐mL initiation injection. Recommendations based on the delay of the 2nd injection are applicable to subsequent injections because exposure prior to the 2nd injection is similar to or lower than subsequent injections.

**Figure 2:**
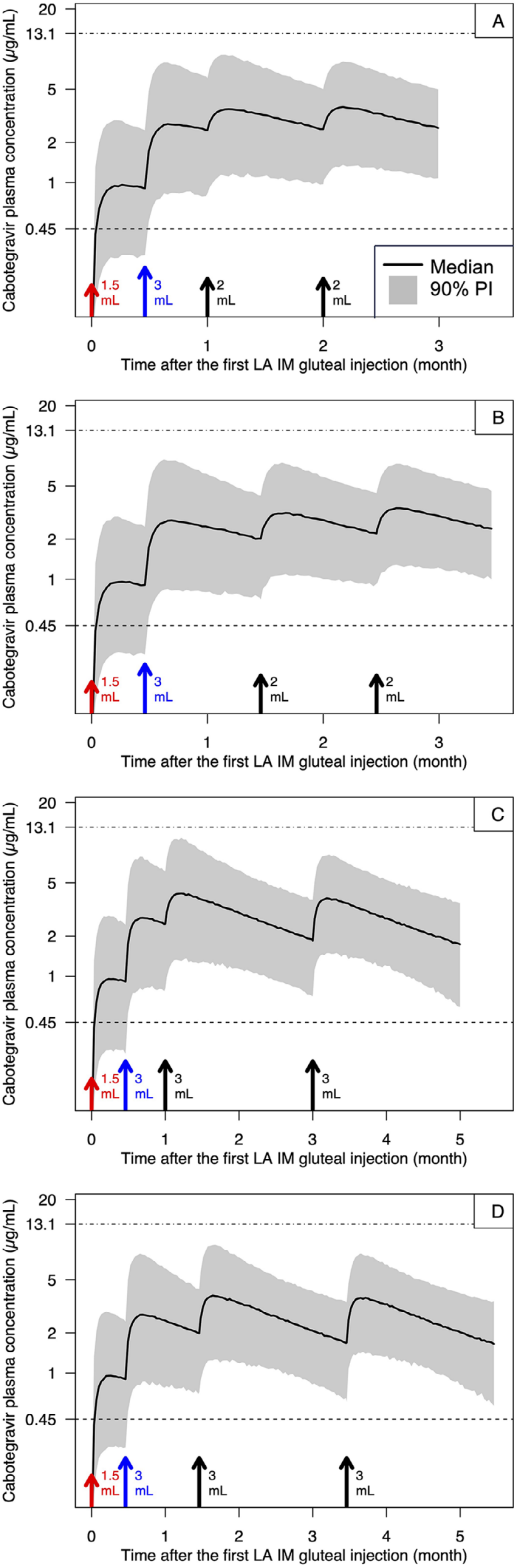
Simulated concentration‐time profiles following lower‐than‐planned doses at the first injection of the QM (A and B) or Q2M (C and D) regimen. Red arrows indicate the incorrect dose of 1.5 mL instead of the planned 3 mL. Blue arrows indicate the corrective injection with the originally planned dose (3 mL) administered 2 weeks after the incorrect dose. Black arrows indicate subsequent injections under the 2 scenarios: (A and C) maintaining the original injection dates or (B and D) resetting the injection dates. The lengths of the arrows and the numbers next to the arrows correspond to the dose level. IM, intramuscular; LA, long‐acting; PI, prediction interval; QM, monthly; Q2M, once every 2 months

Oral bridging was simulated when the 3rd injection was delayed by 1 month (Figure 1G,H) or 2 months (Figure 1I,J). Oral bridging consisted of a CAB 30‐mg oral tablet daily from the day of the missed 3rd injection until the day of the resumed injection. Recommendations based on the 3rd injection are applicable to subsequent injections because exposure following the 3rd injection is similar to or lower than subsequent injections.

### Simulation Methodology

For each scenario described above, CAB concentration‐time profiles were simulated in 5000 virtual subjects (50% female) using the previously established CAB PPK model.^18^ Covariates that were significant in the final model (ie, sex at birth, body weight, body mass index [BMI], and smoking status) were simulated using the relationships between these covariates that were established using the PPK model‐building dataset. Individual PK parameters of the virtual subjects were calculated using subject‐specific covariates and using the population parameter estimates, subject‐specific inter‐individual errors (ETA) sampled from the distributions that are decided by the estimated variance‐covariate matrix of inter‐individual variability from the final PPK model. Residual variability was incorporated in the simulations. All simulations and analyses were performed using R (www.R‐project.org) version 3.6.3.

The CAB PPK model from Han et al^18^ was selected for the simulations based on its comprehensive data foundation, methodological rigor, and robustness. It was developed using data from 1647 individuals and 23,926 plasma concentrations across 16 clinical studies, incorporating extensive covariate assessment. It includes both intensive and sparse PK sampling, both adults with HIV (72%) and without HIV (28%), and both oral tablet and IM gluteal injection. The model spans a wide range of 7 dose levels, including oral doses from 10 to 60 mg and LA doses from 100 to 800 mg. It includes multiple dosing regimens such as daily oral dosing and QM, Q2M, and every 12 weeks (Q12W) LA dosing. The model was externally validated by adequately predicting 5097 concentrations from 647 additional participants who were not used in model development. The structural model was a 2‐compartment model with first‐order absorption and elimination for both routes of administration.

### Adequate CAB Exposure

Simulated CAB exposure was considered likely to maintain efficacy and safety if the trough plasma concentration at the end of the dosing interval immediately prior to the next injection (C_tau_) exceeded the phase 3 benchmark of 0.45 µg/mL in >95% of subjects and the maximum concentration (C_max_) remained below the safety threshold of 13.1 µg/mL in >50% of subjects.

An exposure‐response relationship between CAB plasma PK parameters and efficacy endpoints (virologic response for treatment and seroconversion for PrEP) is challenging to establish due to the low rate of virologic failure for treatment^12^, ^13^, ^14^ and the low rate of seroconversion for PrEP^15^, ^16^ in pivotal studies. Consequently, the phase 3 benchmark was proposed as a tentative probabilistic efficacy target, which was defined as the 5th percentile of the observed C_tau_ after the first injection (C_tau_‐1) observed in the pivotal studies. The rationale was that all phase 3 studies demonstrated robust efficacy from the first injection onward, indicating that C_tau_‐1 was efficacious. Notably, a multivariable analysis by Orkin et al^19^ demonstrated that lower C_tau_‐1 was significantly associated with an increased risk of confirmed virologic failure in CAB+RPV LA recipients, and was selected over steady‐state C_tau_ in the final model, supporting the use of C_tau_‐1 as a surrogate threshold for adequate exposure.^20^ The phase 3 benchmark (5th percentile of observed C_tau_‐1) was 0.45 µg/mL. The adequate outcome was that the 5th percentile of the simulated C_tau_ exceeded the 5th percentile of observed C_tau_‐1 (0.45 µg/mL), that is, C_tau_ exceeded 0.45 µg/mL in >95% of subjects.

The safety threshold was defined as the observed median steady‐state C_max_ of 13.1 µg/mL following daily oral CAB 60 mg (twice the approved CAB oral dose of 30 mg daily) observed in study LAI116482 (NCT01641809),^10^ which was the highest C_max_ ever observed in CAB long‐term studies in adults. This safety threshold was not associated with any toxicity. The adequate outcome was that the median simulated C_max_ remained below the median observed C_max_ (13.1 µg/mL), that is, C_max_ remained below 13.1 µg/mL in >50% of subjects.^20^

All studies complied with the Declaration of Helsinki and Good Clinical Practice guidelines and were approved by the Institutional Review Board (IRB) of participating institutions. All study participants provided written informed consent.

## Results

### Higher‐Than‐Planned Doses

Simulated concentration‐time profiles following the standard CAB QM and Q2M regimens without any dosing deviations or interruptions are displayed in Figure S1. After administering doubled doses at the initiation injection, QM steady‐state injection, and Q2M steady‐state injection, the median C_max_ was predicted to be 4.61, 5.90, and 6.35 µg/mL, respectively (Figure S2), all of which remained well below the safety threshold of 13.1 µg/mL.

### Lower‐Than‐Planned Doses

When the dose of the first injection was halved (red arrows in Figure 2), the 5th percentile of C_tau_ following the incorrect dose was predicted to fall below the benchmark of 0.45 µg/mL (blue arrows in Figure 2). However, when a corrective injection was administered 2 weeks after the incorrect dose, all subsequent C_tau_ values were predicted to return to levels likely to maintain efficacy, regardless of maintaining the original injection dates (Figure 2A,C) or resetting the injection dates (Figure 2B,D).

When the dose of the 2nd injection was halved, C_tau_ was predicted to remain above levels likely to maintain efficacy before and after the corrective injection was administered, regardless of maintaining the original injection dates (Figure S3A,C) or resetting the injection dates (Figure S3B,D).

### Tolerable Injection Window

**Figure 3:**
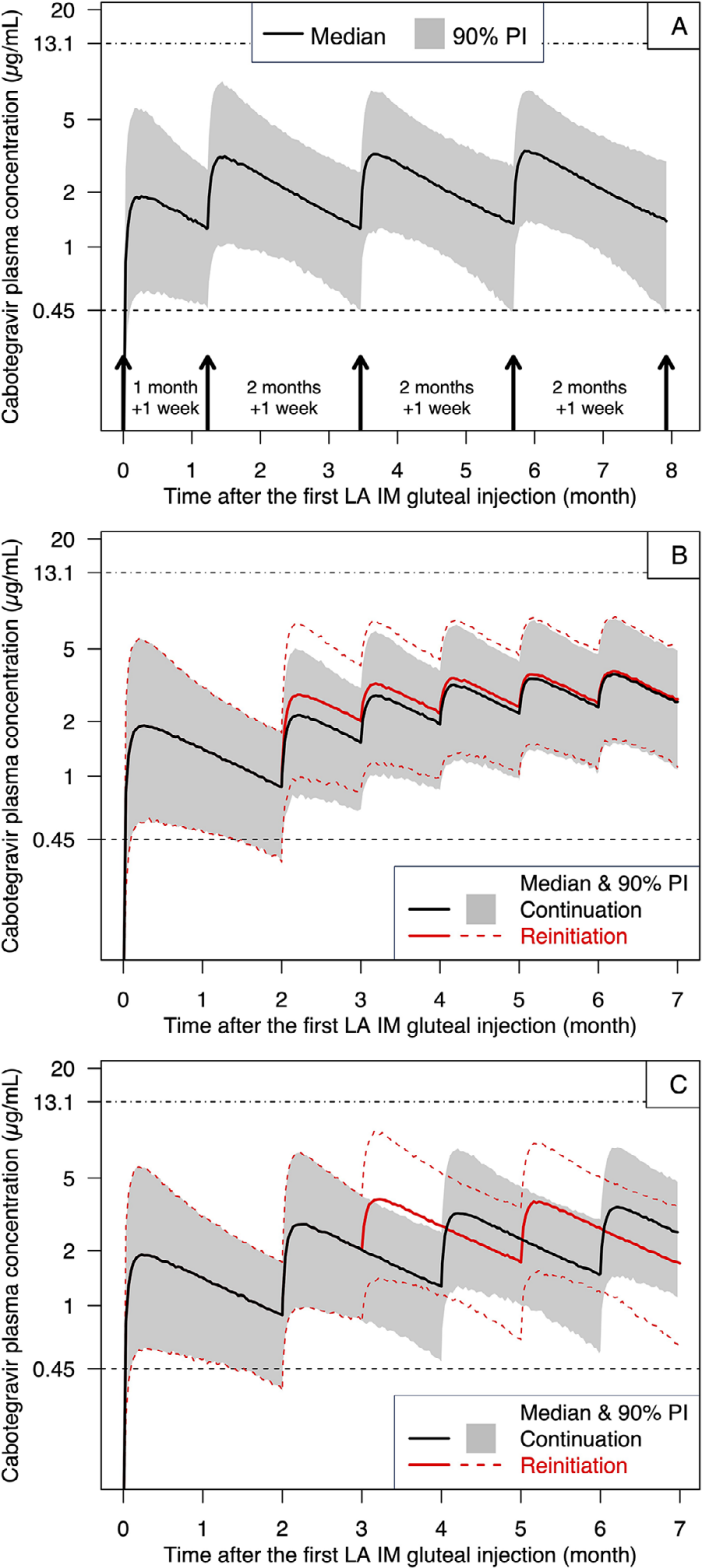
Simulated concentration‐time profiles following delayed injections: every injection of the Q2M regimen was delayed by 7 days (A), or the 2nd injection of the QM (B) or Q2M (C) regimen was delayed by 1 month. On resumption of the delayed injection after a 1‐month delay (B and C), continuation of regular QM (B) or Q2M (C) maintenance injections was compared to reinitiation of the QM (B) or Q2M (C) regimen. Continuation (black line and grey band in B and C) was defined as resumption of regular maintenance dose (2 mL QM or 3 mL Q2M) after the delay. Reinitiation (red solid and dashed lines in B and C) was defined as resumption with a 3‐mL initiation injection, followed by 2 mL QM (QM regimen) or 3 mL Q2M (Q2M regimen) starting 1 month after the 3‐mL initiation injection. IM, intramuscular; LA, long‐acting; PI, prediction interval; QM, monthly; Q2M, once every 2 months.

Under the extreme scenario where every injection of the Q2M regimen was delayed by 7 days, C_tau_ following every injection was predicted to remain above levels likely to maintain efficacy (Figure 3A).

### Unplanned Dosing Interruptions

Longer delays led to a prolonged period below the probabilistic efficacy target prior to resuming the delayed injection (Table S1). Delays of >7 days were predicted to result in suboptimal exposure prior to resuming the delayed injection. The longest simulated delay was a delay of 3 months (ie, 2 maintenance injections in the QM regimen were 4 months apart instead of 1 month apart, or 2 maintenance injections in the Q2M regimen were 5 months apart instead of 2 months apart). In the 3‐month delay scenario, C_tau_ was predicted to remain below the probabilistic efficacy target for up to 83 days.

For the QM regimen, delaying an earlier injection led to lower CAB exposure before resuming the delayed injection than delaying later injections (Table S1) because of the accumulation of CAB exposure following later injections. CAB exposure following the QM regimen was predicted to remain above the probabilistic efficacy target if the 2nd injection was delayed by up to 7 days, the 3rd injection was delayed by up to 3 weeks, or the 4th injection was delayed by up to 4 weeks. In contrast, CAB exposure in the Q2M regimen was predicted to be similar regardless of which injection was delayed due to the lack of accumulation.

When the delayed injection was resumed, all subsequent C_tau_ values were predicted to return to levels likely to maintain efficacy, regardless of continuation (ie, regular maintenance dose 2 mL QM or 3 mL Q2M was resumed after the delayed injection) or reinitiation (ie, 3‐mL initiation injection was resumed after the delayed injection, followed by 2 mL QM or 3 mL Q2M starting 1 month after the initiation injection). Following the resumed injection, reinitiation led to higher C_tau_ than continuation (Figure 3B,C).

### Planned Dosing Interruptions With Oral Bridging

**Figure 4:**
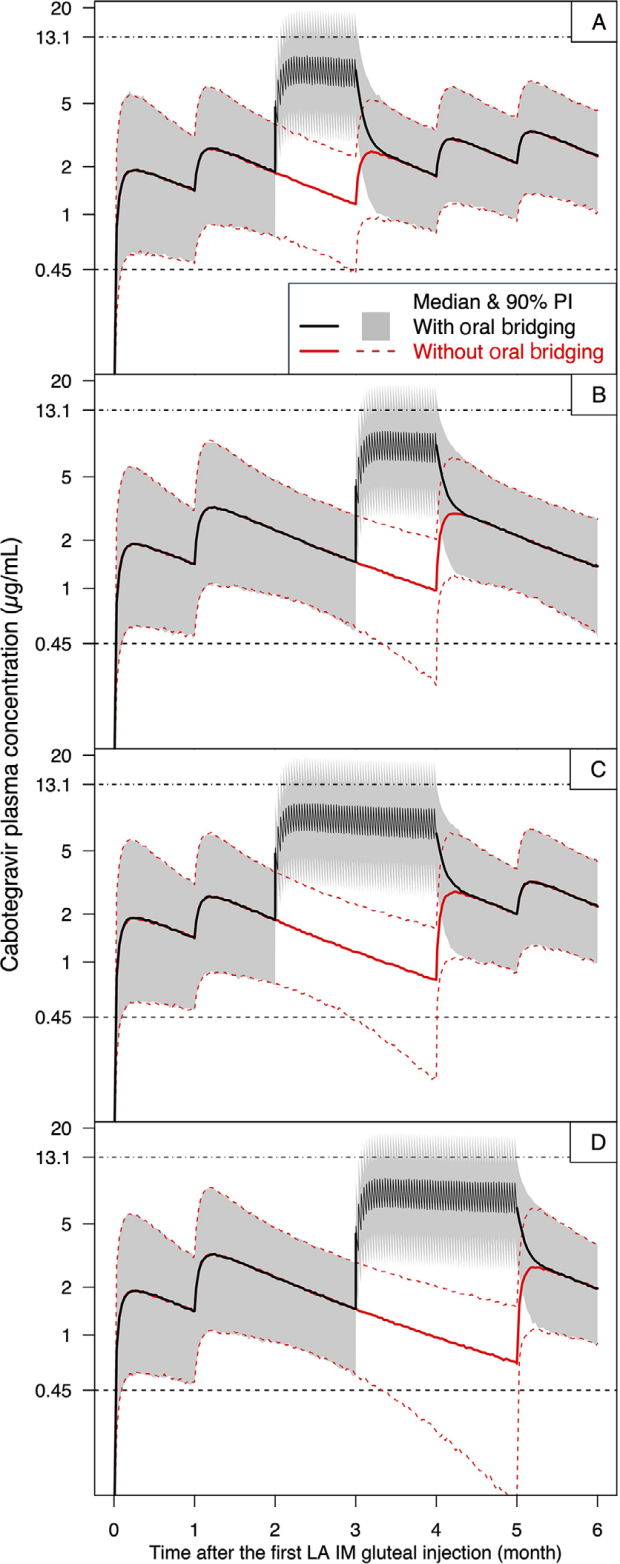
Simulated concentration‐time profiles following oral bridging for 1 month with continuation on resuming the delayed injection (A and B) or 2 months with reinitiation on resuming the delayed injection (C and D) in the QM (A and C) and Q2M (B and D) regimens. Oral bridging consisted of cabotegravir 30 mg oral tablet daily from the day of the missed injection until the day of the resumed injection. Continuation was defined as resumption of regular maintenance dose (2 mL QM or 3 mL Q2M) after the delay. Reinitiation was defined as resumption with a 3‐mL initiation injection, followed by 2 mL QM (QM regimen) or 3 mL Q2M (Q2M regimen) starting 1 month after the 3‐mL initiation injection. IM, intramuscular; LA, long‐acting; PI, prediction interval; QM, monthly; Q2M, once every 2 months.

Planned dosing interruptions without oral bridging resulted in outcomes similar to those of unplanned dosing interruptions as described above. Following a 1‐month delay of the 3rd injection without oral bridging, the 5th percentile of C_tau_ was predicted to remain below the phase 3 benchmark of 0.45 µg/mL for 2 days for the QM regimen (Figure 4A and Table S1) and 23 days for the Q2M regimen (Figure 4B and Table S1). Following a 2‐month delay of the 3rd injection without oral bridging, the 5th percentile of C_tau_ was predicted to remain below the phase 3 benchmark of 0.45 µg/mL for 32 days for the QM regimen (Figure 4C and Table S1) and 53 days for the Q2M regimen (Figure 4D and Table S1). However, when oral bridging was implemented, both C_tau_ and C_max_ were predicted to remain within exposure ranges likely to support efficacy and safety.

## Discussion

This analysis highlights the use of modeling and simulations to develop CAB dosing strategies and gain regulatory approvals, circumventing the need for dedicated clinical studies. The impact and corrective actions of higher‐than‐planned doses, lower‐than‐planned doses, and delayed injections with or without oral bridging were assessed using PPK simulations. Although no new data were generated in this analysis, the findings informed label languages and prescribing information in the sections of recommended injection dosing, overdose management, and recommended dosing schedule for missed injections of CAB LA and RPV LA. All recommendations developed for CAB LA align with those for managing injection delays of RPV LA.^21^, ^22^ This experience can benefit future clinical development of next‐generation CAB formulations^23^ and other LA agents, facilitating more efficient clinical development and regulatory decision‐making.

It is challenging and impractical to conduct clinical studies with the sole purpose of assessing the impact and corrective actions of dosing deviations and interruptions for LA injectables. The long half‐life and extended residence time of LA agents require prolonged studies, which can be prohibitively expensive and time‐consuming while offering limited benefit to participants. Modeling and simulations provide an instructive alternative, especially given the robustness of the CAB PPK model,^18^ which was established based on 23,926 plasma concentrations from 1647 adults with HIV (72%) and without HIV (28%) in 16 clinical studies and validated by adequately predicting 5097 plasma concentrations from 647 additional participants who were not included in the model‐building dataset.

For scenarios involving higher‐than‐planned doses, no corrective action is required and dosing may continue as planned. Under the extreme scenarios of doubled doses at steady state, CAB C_max_ was predicted to be >50% below the safety threshold. The elevated exposure was primarily confined to the dosing interval immediately following the higher‐than‐planned dosing event and rapidly returned to normal levels. This is fortunate because eliminating an excess dose of CAB is challenging due to several factors: (1) extended washout period; (2) CAB is highly bound to plasma proteins and therefore unlikely to be efficiently removed by dialysis; and (3) for the treatment regimen with CAB+RPV, any action to remove CAB might also inadvertently remove RPV while RPV may have been correctly administered and therefore does not warrant removal.

Lower‐than‐planned doses were predicted to result in suboptimal CAB C_tau_ without a corrective injection if the incorrect dose was the first injection for both regimens (Figure 2) or any injection of the Q2M regimen. Therefore, administering a corrective injection as soon as feasible may prevent further decline in concentrations, which could jeopardize efficacy and increase the risk of resistance. Due to the high safety threshold, the dose of the corrective injection can be the full dose. The timing of the corrective dose is flexible: even if the corrective injection is administered immediately after the incorrect dose, there is no concern of overdose because even doubled doses would lead to a C_max_ well below the safety threshold.

In clinical practice, lower‐than‐planned doses may not always be apparent to the administering clinicians. However, if a dosing deviation is suspected, such as visible seepage, a confirmed vial mismatch, or an interrupted injection (eg, due to pain, injection resistance, or device malfunction), a full corrective dose may be administered as soon as feasible. Even if the original dose was fully delivered and the corrective injection was administered unnecessarily, there is no concern of overdose because even doubled doses would lead to a C_max_ well below the safety threshold.

After the corrective injection is administered, subsequent injections can start 1 month (QM regimen) or 2 months (Q2M regimen) either after the incorrect dose (ie, maintaining the original injection dates) or after the corrective injection (ie, resetting the injection dates). Both approaches were predicted to result in adequate CAB exposure, although the former approach is preferable for the treatment regimen with CAB+RPV if only the CAB dose was lower than planned, while RPV was correctly administered.

Adherence to the prescribed QM or Q2M dosing schedule is recommended. The purpose of the tolerable injection window is to provide some flexibility in scheduling. The tolerable injection window was predicted to be ±7 days as C_tau_ was predicted to remain above the probabilistic efficacy target under the extreme scenario that every injection of the Q2M regimen was delayed by up to 7 days. Simulations of –7 days are not displayed because an injection administered 7 days earlier than scheduled would lead to higher exposure.

Avoiding dosing interruptions and delays may help maintain therapeutic exposure levels. If the 2nd injection of either regimen was delayed by >1 month, underexposure was predicted to last >18 days. If the 3rd injection or later injections of the Q2M regimen were delayed by >1 month, underexposure was predicted to last >23 days. Due to this prolonged underexposure, it is recommended to reinitiate the loading dose after resuming the injection that was delayed by >1 month to reduce the risks of reduced efficacy and resistance development because reinitiation with the loading dose led to higher exposure than continuation with regular maintenance doses (Figure 3). To simplify clinical practice and avoid confusion, the same approach for managing injection delays was considered appropriate for both QM and Q2M regimens, regardless of whether oral bridging is used.

For subgroups stratified by sex, BMI, and smoking status, simulations were repeated using subgroup‐specific target exposures, and the conclusions and recommendations remained unchanged. For example, females have a longer half‐life and therefore a higher steady‐state C_tau_ than males. As a result, a consistent 1‐month delay of the Q2M regimen (ie, Q3M dosing) in females yields C_tau_ similar to that of males receiving on‐time Q2M dosing. Despite comparable C_tau_, female Q3M dosing cannot be recommended without empirically observed data^24^ because efficacious exposure for the Q2M regimen for PrEP was established separately for men and women.

The phase 3 benchmarks cited in previous publications^25^ (0.65 µg/mL) and in this report (0.45 µg/mL) are consistent. These 2 values are the 5th percentile of the individual predicted values (IPRED) and the observed values (OBS) of C_tau_‐1, respectively. IPRED contains no residual variability (eg, assay errors) and, accordingly, simulations in previous publications were conducted without residual variability and therefore compared to 0.65 µg/mL.^25^ In contrast, simulations in this report were conducted with residual variability and therefore compared to 0.45 µg/mL. The initial exploration was performed using IPRED values because the observed data were still being collected and cleaned at that time. However, it was always the intention to repeat the analyses by incorporating residual variability and to compare them against observed benchmarks once the full dataset became available. This approach allows for a more realistic representation of real‐world variability. As expected, the simulation outcomes and the resulting recommendations are consistent across both approaches. In addition, thresholds such as 1×, 4×, or 8× in vitro protein‐adjusted concentration resulting in 90% of the maximum inhibition (PA‐IC90) of viral growth for CAB were used during early development when clinical data were limited, but the focus shifted to the empirically derived clinical benchmark of 0.45 µg/mL once sufficient phase 3 clinical data became available.

There are several limitations to this analysis. First, dose proportionality up to 6 mL (1200 mg) was assumed, while no observed CAB PK data are available at this dose level. Second, simulation outcomes under some of the scenarios have not been confirmed in clinical settings. Real‐world experience and data from ongoing clinical studies of CAB may provide additional evidence to further validate the simulation outcomes. Third, these dosing strategies are based on simulations while actual CAB concentrations may vary substantially between individuals, as reflected by the wide prediction intervals in the simulated profiles, therefore the recommendations should be viewed as initial guidance rather than precise correction tools. Clinical judgment and ongoing monitoring of patient outcomes remain essential.

## Conclusions

For higher‐than‐planned CAB LA doses of up to 6 mL (1200 mg), no corrective action is required. For lower‐than‐planned CAB LA doses, it is recommended to administer a corrective injection as soon as practically possible. Adherence to the QM or Q2M dosing schedule is recommended. The tolerable injection window is ±7 days. If a CAB LA injection is delayed by ≤1 month (QM regimen: ≤2 months between injections; Q2M regimen: ≤2 months between the first 2 injections or ≤3 months between subsequent injections), it is recommended to resume regular maintenance injections (2 mL QM or 3 mL Q2M) regardless of whether oral bridging is used. If a CAB LA injection is delayed by >1 month (QM regimen: >2 months between injections; Q2M regimen: >2 months between the first 2 injections or >3 months between subsequent injections), it is recommended to reinitiate the regimens (QM regimen: 3‐mL initiation injection followed by 2‐mL maintenance injections QM; Q2M regimen: 3‐mL initiation injection followed by 3‐mL maintenance injections Q2M starting 1 month after the resumed 3‐mL initiation injection) regardless of whether oral bridging is used. Daily oral administration of a 30‐mg CAB tablet can maintain therapeutic CAB exposure during planned interruptions. Although no new data were generated in this analysis, the findings were incorporated into the product labels and prescribing information of CAB LA for HIV treatment and PrEP. This analysis demonstrates the value of modeling and simulations in optimizing dosing strategies for CAB LA without requiring dedicated clinical studies. The approaches outlined here can inform the future development of next‐generation CAB formulations and otherLA agents.

## Conflicts of Interest

Kelong Han and Susan L. Ford receive salary from GSK and hold stocks of GSK. Ronald D. D'Amico and William R. Spreen receive salary from ViiV Healthcare and hold stocks of GSK.

## Funding

This research received no specific grant from any funding agency in the public, commercial, or not‐for‐profit sectors.

## Supporting information



Supporting Information
