# Supplementary material for: Strategies to Manage Dosing Deviations and Interruptions of Cabotegravir Long‐Acting Intramuscular Injections
Source: Clin Pharmacol Drug Dev. 2025 Jul 11;15(1):e1568. doi: 10.1002/cpdd.1568 (PMC12814316; doi:10.1002/cpdd.1568)

SUPPLEMENTAL MATERIALS

Table S1. Duration (days) of inadequate exposure before resuming the delayed injection. Exposure was considered adequate if the trough concentration exceeded the phase 3 benchmark of 0.45 µg/mL in >95% of subjects.

Regimen	Delayed Injection	Length of Delay											
		1 wk	2 wk	3 wk	4 wk	5 wk	6 wk	7 wk	8 wk	9 wk	10 wk	11 wk	12 wk
Both	2nd injection	0	3	10	18	25	33	40	48	55	63	70	78
QM	3rd injection	0	0	0	2	9	17	24	32	39	47	54	62
QM	4th injection	0	0	0	0	6	14	21	29	36	44	51	59
Q2M	3rd injection	0	8	15	23	30	38	45	53	60	68	75	83

Both: both QM and Q2M regimens because the first injection and the time of the second injection are the same for both QM and Q2M regimens; QM: once monthly; Q2M: once every 2 months; wk: week(s). The 4th injection of the Q2M regimen is not displayed because the values are identical to the 3rd injection. The heatmap spectrum ranges from green (fewer days) to red (more days).

Figure S1. Simulated concentration-time profiles following cabotegravir long-acting injections in the approved QM (A) and Q2M (B) regimens consisting of a 3 mL (600 mg) initiation injection followed by maintenance injections of 2 mL (400 mg) QM (QM regimen) or 3 mL (600 mg) Q2M (Q2M regimen) starting 1 month after the initiation injection. IM, intramuscular; LA, long-acting.

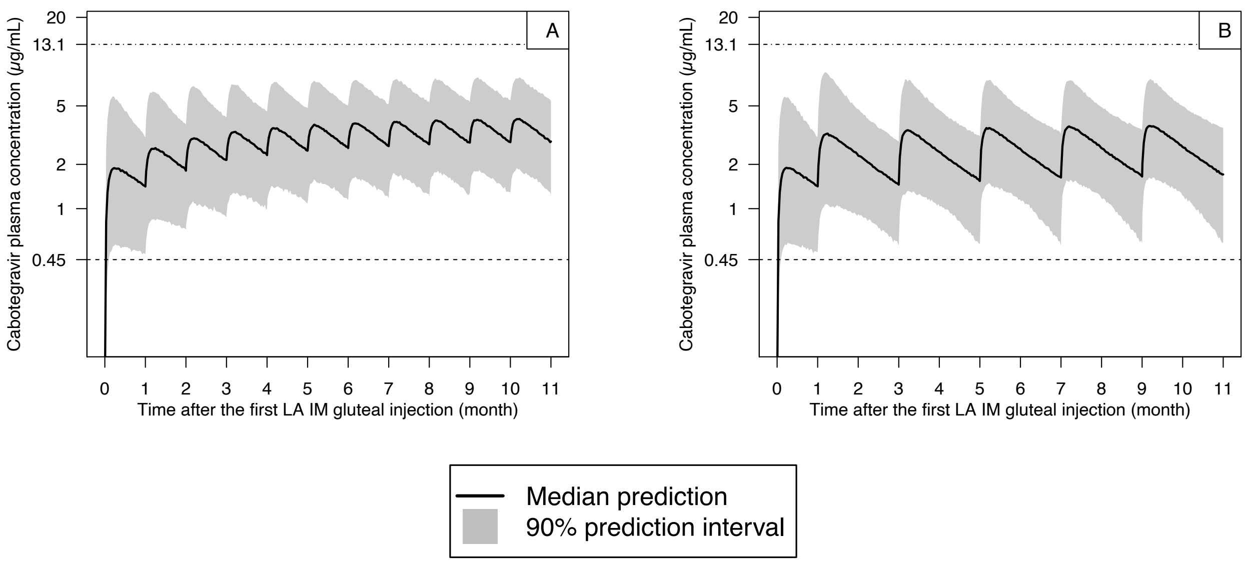

Figure S2. Simulated concentration-time profiles following higher-than-planned dose for the QM regimen (A and B) and the Q2M regimen (C and D) at the first injection (A and C) and at steady state (B and D). Red arrows indicate the incorrect dose of 6 mL or 4 mL instead of the planned 3 mL or 2 mL, respectively. Black arrows indicate injections with the originally planned doses. The lengths of arrows and the numbers next to the arrows correspond to the dose level. IM, intramuscular; LA, long-acting.

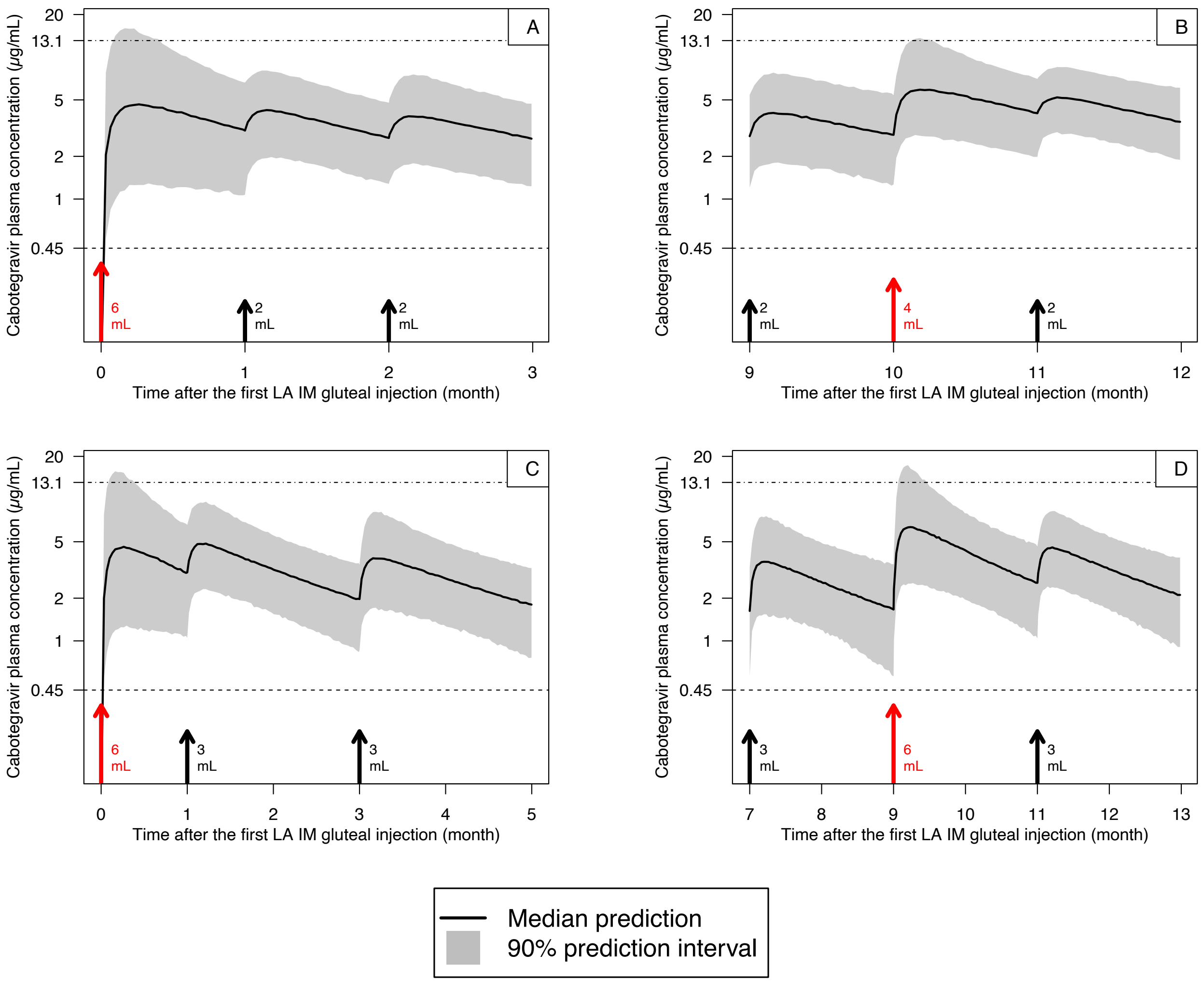

Figure S3. Simulated concentration-time profiles following lower-than-planned dose at the 2nd injection of the QM (A and B) or Q2M (C and D) regimen. Red arrows indicate the incorrect dose of 1 mL or 1.5 mL instead of the planned 2 mL or 3 mL, respectively. Black arrows indicate subsequent injections under the 2 scenarios of maintaining the original injection dates (A and C) or resetting the injection dates (B and D). The lengths of arrows and the numbers next to the arrows correspond to the dose level. IM, intramuscular; LA, long-acting.

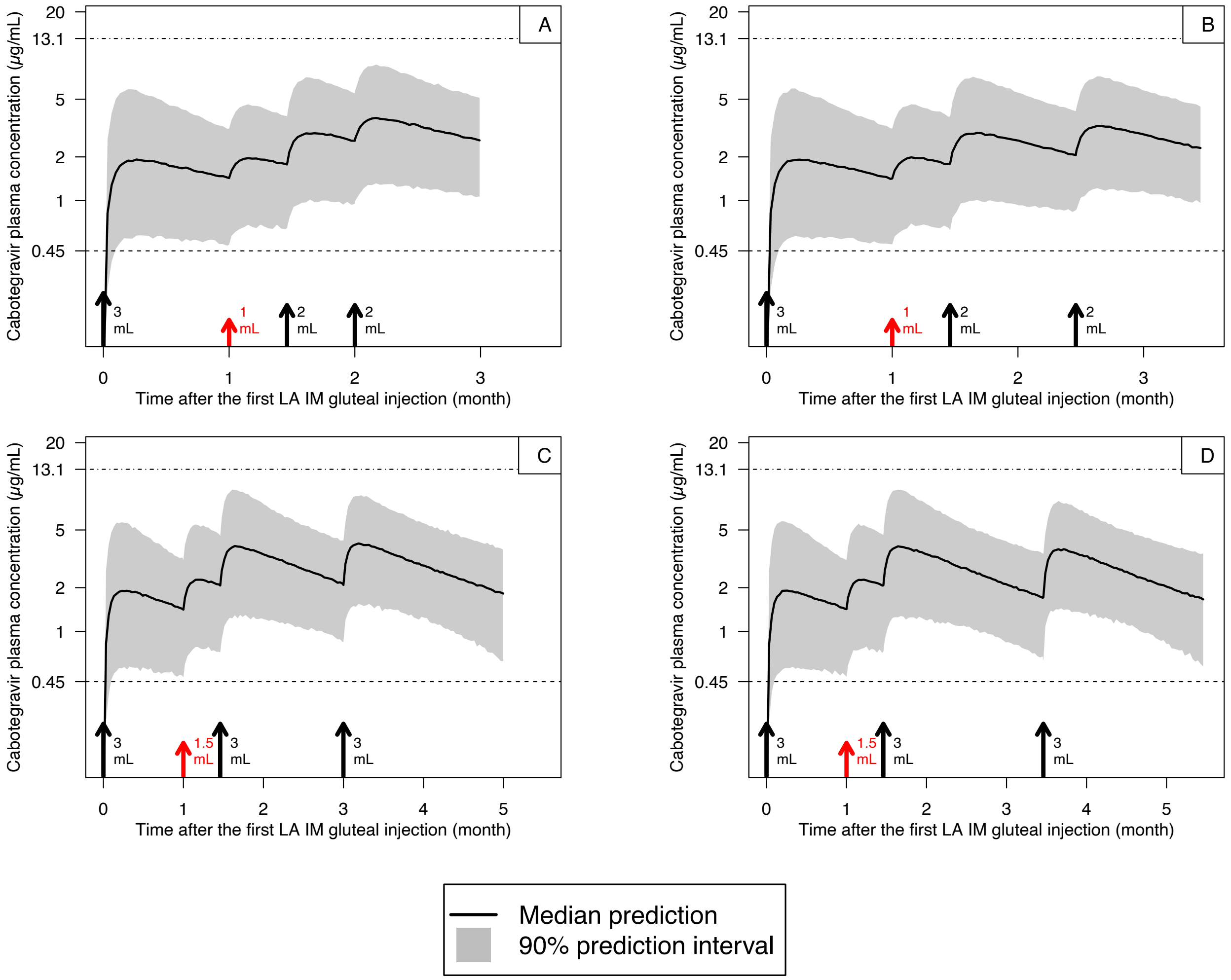

Supplement: Supplementary file 1 — Supporting Information [file CPDD-15-0-s001.pdf]
